# Clinical-Functional Vulnerability, Functional Capacity, and Falls in Octogenarians with Different Physical Activity Levels—A Cross-Sectional Study

**DOI:** 10.3390/ijerph191911909

**Published:** 2022-09-21

**Authors:** Letícia Pophal da Silva, Natália Boneti Moreira, Renata Zacharias Grando, Paulo César Baraúce Bento, André Luiz Felix Rodacki

**Affiliations:** 1 Department of Physical Education, Federal University of Paraná, Curitiba 80310-000, PR, Brazil; 2 Department of Prevention and Rehabilitation in Physical Therapy, Federal University of Paraná, Curitiba 80310-000, PR, Brazil

**Keywords:** frailty, functional performance, falls, oldest-old adults, octogenarians

## Abstract

Aim: To compare differences between frailty, functional capacity, and fall prevalence among community-dwelling oldest-old adults regarding their physical activity levels. Methods: Two hundred and thirty-nine octogenarians (80+ years) were allocated according to their physical activity as insufficiently active (<150 min week^−1^; *n* = 98; 84.4 ± 3.7 years), active (150 to 300 min week^−1^, *n* = 81, 83.9 ± 3.1 years), and very active (>300 min week^−1^, *n* = 60; 83.8 ± 3.4 years). Frailty (CFVI-20 questionnaire), functional capacity (Five Times Sit-to-Stand Test, Timed Up and Go, Balance, and handgrip strength), fall history, and physical activity were assessed. Results: The insufficiently active group was the frailest and presented the worst functional performance compared to the other groups. The fall prevalence was higher in the insufficiently active (60.9%) compared to the active (26.4%) and very active (12.7%) groups. Conclusions: The group of insufficiently active octogenarians showed the greatest frailty, worst functional capacity, and higher fall prevalence than the active and very active groups. The engagement in physical activity of at least 300 min week^−1^ is essential to reverse or minimize the deleterious effects of aging on frailty, functional capacity, and falls in octogenarians.

## 1. Introduction

Aging has been associated with an increase in morbidity and death and represents a major socioeconomic problem faced by several countries. Brazil has the fifth largest population in the world, with over 230 million inhabitants, from which more than one-third (33.7%) will be aged 60 or more in 2060. Given the growth in the number of older adults and the increases in life expectancy, it is necessary to pay special attention to the oldest-old adults (80 and older). The number of health problems is closely associated with longevity, and the oldest-old are likely to experience more pronounced changes.

The natural age-related declines in physical and functional performance combined with a sedentary lifestyle (i.e., a low level of physical activity) have been associated with increased frailty [1]. Frailty is characterized by progressive loss of strength, endurance, and decreased physiological responses, which increases vulnerability, functional disabilities, frequent hospitalization, and mortality when exposed to a stressor [2]. Frailty has also been associated with fall prevalence. Moreira and colleagues reported that frail older adults have a 5.8 times more risk of experiencing a fall compared to robust counterparts—i.e., with low vulnerability to frailty [3]. In addition, frailty and falls have been associated with reduced functional capacity in older adults, as revealed by poor performance in several physical tests (e.g., muscle strength, balance, walking speed, etc.). Interestingly, frailty, functional impairments, and falls are described as positively impacted by physical activity increments (PA) [4].

Regular PA is a significant health marker that affects functional capacity, risk of falling, and frailty in older adults [4,5,6]. In recent years, increased PA and exercise programs have been proposed as a preventive strategy for frailty and its adverse outcomes, as they can target several frailty criteria aspects. With advancing age, the oldest-old tend to decrease PA levels, and reversing such a trend represents a global growing public health challenge. On the other hand, lifestyle changes emphasizing physically active behaviors have been widely encouraged as an essential component when the adverse effects of aging are to be minimized or postponed [7].

Most studies on the effects of physical activity on aging have focused on young or middle older adults [8], while the oldest-old adults have received less attention, especially regarding functional capacity, frailty, and fall risk. Additionally, oldest-old adults with a very active physical activity profile may have improved functional capacity, lower vulnerability to frailty, and reduced fall prevalence compared to those with an inactive physical activity profile. Therefore, very active oldest-old adults (>300 min week^−1^) may have less pronounced adverse effects on frailty, functional capacity, and fall prevalence than those physically less active (i.e., <150 min week^−1^). Thus, this study aimed to compare frailty, functional capacity, and fall prevalence among oldest-old adults (80+ years) according to their PA levels (i.e., insufficiently active, active, and very active).

## 2. Methods

This cross-sectional study was conducted with community-dwelling oldest-old adults in Curitiba, Brazil, a city of 1,751,907 inhabitants, from which 1.56% (*n* = 27.244) are aged 80 years or older. The sample size was calculated using the Epi info calculator developed by the Center for Disease Control and Prevention using the following parameters: (i) population of 27,244 individuals 80 years or older from the city of Curitiba, Paraná, Brazil, (ii) 80% power, (iii) sampling error of 5%, (iv) 50% of anticipated frequency, considering the maximum variance, (v) design effect of 1.0 to correct the sample selection biases, and (vi) 20% margin for possible losses and refusals. Therefore, the estimated sample size was 197 participants.

The participants were 80 years or older, of both sexes, and capable of walking independently and without assistive devices. The exclusion criteria were: (i) Neurological disease, (ii) musculoskeletal problems that limited their accomplishment in all procedures, (iii) unstable and severe health conditions, (iv) mobility deficits that prevent the older adults from moving in the orthostatic position independently, and (v) cognitive impairment that prevents the participant from understanding the assessment, via the Mini-Mental State Examination.

**Figure 1:**
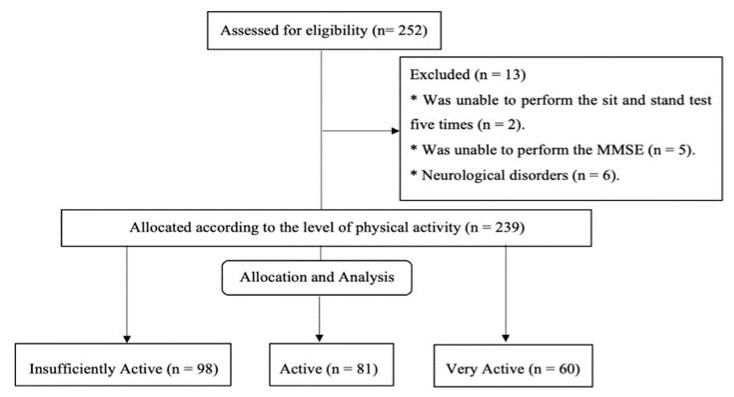
The flow diagram of the groups represents the allocation of Insufficiently Active, Active, and Very Active groups according to their level of physical activity (PA).

Then, 239 oldest-old (176 older women—74% and 63 men—26%) agreed to participate by signing an informed consent form. The Research Ethics Committee of the University (process number: 48548715.5.0000.5223) and the Curitiba County Health Board (process number: 48548715.5.3001.0101) approved all study procedures. Figure 1 shows a schematic representation of the group allocation.

The participants completed a 40 to 60 min assessment session composed of questions related to sociodemographic characteristics (i.e., age, gender, body mass index (BMI), educational level, and marital status), PA level, history of falls, functional capacity, and the clinical-functional vulnerability questionnaire (CFVI-20).

The Minnesota Leisure Time Activities Questionnaire was applied to assess the PA level. The instrument is widely used in older adults and was previously validated for the country population [9]. The weekly volume (min per week) of PA was used to classify the sample as insufficiently active (<150 min/week), active (150 to 300 min/week), and very active (>300 min/week), according to the American College of Sports Medicine [10]. The Clinical-Functional Vulnerability Index-20 (CFVI-20) is a questionnaire that includes multidimensional aspects of the condition of older adults and is a frailty screening tool. The higher the score, the greater the risk of clinical and functional vulnerability [11].

The functional tests were performed in a standard order, and three to five minutes of rest was imposed between tests. Participants completed a familiarization trial for each test. The functional tests included: Lower limb muscle power (Five Times Sit-to-Stand Test; 5STS), functional mobility (Timed Up and Go; TUG), balance (Berg Balance Scale; BBS), and strength (Hand grip strength; HGS, Jamar^®^, Sammons Preston Rolyan, Chicago, IL, USA). The test-retest reliability of the functional tests (5STS, ICC = 0.96; TUG, ICC = 0.96; BBS, ICC = 0.87, and HGS, ICC ≥ 0.85) and PA level (Minnesota, ICC = 0.79–0.88) are reported as high.

Fall episodes were assessed by asking participants whether they had experienced a fall during the last 12 months. A fall was defined as an unintentional event that resulted in changing the position to a lower level relative to his/her initial position, irrespective of whether an injury was caused. Those who reported a fall in the last twelve months were deemed as fallers regardless of the number of events.

Descriptive statistics (mean and standard deviation) were performed to characterize the groups. Data normality was tested using the Kolmogorov–Smirnov, and non-normal distribution data were treated using non-parametric tests. The groups (IAG vs. AG vs. VAG) were compared using the Kruskal–Wallis and Chi-Square tests according to the analyzed variable. The Bonferroni test was applied to identify where differences occurred (*p* < 0.01). To observe the association between PA and functional capacity (i.e., TUG, 5STS, BBS, and HGS) and frailty vulnerability (i.e., CFVI-20) of fallers and non-fallers, several linear regression analyses were performed. The statistical procedures were performed in the SPSS software (IBM, version 25, Armonk, NY, USA), and the significance level was set at *p* < 0.05.

## 3. Results

The study comprised 239 community-dwelling octogenarians aged 84.3 ± 3.0 (80 to 96 years). The PA of the total sample was 209 ± 157 min week^−1^. Participants were allocated in the groups according to their PA level: insufficiently active group (IAG, <150 min week^−1^, *n* = 98; 41.0%), active group (AG, 150 to 300 min week^−1^, *n* = 81; 33.9%), and very active group (VAG, >300 min week^−1^, *n* = 60; 25.1%). Most participants were female (73.6%), studied between 1–4 years (52.3%), and predominantly widows (65.3%). The age, BMI, gender, education level, and marital status did not differ among the groups (*p* > 0.05). The sociodemographic characteristics can be seen in Table 1.

Table 2 compares the frailty, functional capacity, and fall history of all groups. The IAG scored highest on the CFVI-20, indicating a more pronounced vulnerability to frailty. Individuals classified as the most vulnerable to frailty (*n* = 74) were predominantly in the IAG group (*n* = 44). In addition, the IAG presented the worst functional capacity scores (5STS, TUG, and BBS) and a higher fall prevalence than the AG and VAG. Most falls (60%) were observed in the IAG, presenting the worst performance compared to all groups in all functional tests. The HGS did not differ between groups.

**Figure 2:**
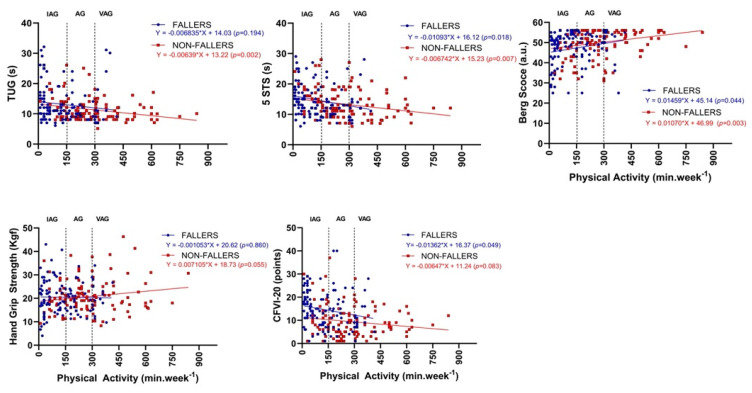
The relationship between physical activity and the functional capacity tests and frailty (CFVI-20) of oldest-old adults with (blue circles) and without (red squares) fall history. Note: The vertical dashed lines indicate the limits for the insufficient (IAG), active (AG), and very active groups (VAG). The blue and red solid lines represent the fitted lines of the linear regression equations. The linear regression equations and significance levels are presented in the legends.

Figure 2 presents the association between PA and functional capacity of oldest-old adults with and without falls. It was observed that most participants with a fall history belong to the IAG. The AG presented a mixed composition of fallers and non-fallers. However, the VAG showed a strong predominance of non-fallers in almost all physical tests. The negative slopes of the TUG, and 5STS, indicate that faster performance occurred in those with greater PA, while the BBS and the HGS tests revealed a positive association. It was observed that a reduction of one second in the TUG requires fallers to increase their PA by 73 min week^−1^ (*p* = 0.194), while the non-fallers by 154 min week^−1^ (*p* = 0.02). Interestingly, a change of one unit in the BBS required the PA to increase by 68 min week^−1^ for the fallers (*p* = 0.044) and by 93 min week^−1^ for the non-fallers (*p* = 0.003). On the other hand, improvements of one unit in the 5STS test demanded 12 min week^−1^ for the fallers (*p* = 0.018) and non-fallers (*p* = 0.007). According to the regression analysis, changes of 1 kgf in HGS could be introduced with no more than 7 min week^−1^ in PA in both groups (fallers *p* = 0.860; non-fallers, *p* = 0.055).

Figure 2 also indicates that increasing PA can reduce frailty vulnerability. The prevalence of frailty in the IAG was prominent, while fall history was scarce in the VAG. It was observed that promoting a one-unit change in frailty vulnerability was possible by increasing the PA by 73 min week^−1^ for the fallers (*p* = 0.049) and 154 min week^−1^ for the non-fallers (*p* = 0.083). The non-fallers showed a non-significant association.

## 4. Discussion

This study was designed to compare frailty, functional capacity, and fall prevalence among community-dwelling oldest-old adults (80+ years and over) with different physical activity levels. The results indicate that those with the lowest PA (IAG) were the most vulnerable to frailty and showed the worst performance in all functional tests (TUG, 5STS, and BBS), while no differences were noted between groups in the hand-grip strength test. In addition, oldest-old adults with insufficient PA (<150 min week^−1^) experienced more falls than their active and very active counterparts.

The group with insufficient PA was classified as frail (14.4 points) and presented a frailty prevalence 2.8 times greater than older adults approximately 15 years younger (i.e., 44.9% vs. 15.8% [3]). These results can be explained by the large impact of the physical activity domain on frailty. Although the CFVI-20 includes several domains (e.g., health self-awareness, cognition, humor, communication, and multiple comorbidities), physical aspects are closely related to functional performance [12]. Indeed, the insufficiently active group showed the worst functional capacity performance compared to the active and very active groups.

PA has been proposed as one of the most effective alternatives to mitigate the adverse effects of aging and reverse frailty [10,13]. Our results support the idea that increasing PA by approximately 73 min week^−1^ reduces the CFVI-20 frailty score by one unit in oldest-old adults with a fall history. The greater PA engagement needed for the non-fallers may be related to the fact that it is more challenging to promote changes in very active older adults than in those with insufficient PA or sedentary status [14].

The overall fall prevalence was 46%, confirming that it increases with advancing age, especially among the oldest-old adults. Moreira and colleagues reported an odds ratio for falls of 1.6% per year for older adults [3], which is within our overall fall prevalence. The overall fall prevalence indicated that 40 to 50% of older adults are likely to fall once a year [15]. These findings reinforce the relevance of PA among oldest-old adults as a potentially protective factor and contradict the understanding that senescence is necessarily accompanied by frailty.

The oldest-old adults with insufficient PA performed the TUG above 13.5 s and presented a higher fall prevalence (68.4%) compared to their physically active peers. However, these cutoffs must be exercised with caution since there are arguments that the TUG test is more useful at ruling in rather than out individuals classified as high-risk [16]. Interestingly, the oldest-old adults with insufficient PA concluded the 5STS above 15 s, which is also above the cutoff reference for falls. Buatois and colleagues proposed that older adults who performed the 5STS above 15 s have a 74% greater risk for recurrent falls [17]. The balance test results also corroborate the argument that insufficient PA is associated with increased fall risk, as they were close to the cutoff applied to identify fallers (<45 or lower) [18].

Although the association between insufficient PA and falls has been relatively well-established, some very active octogenarians experienced a fall. A U-shaped association has been proposed in which very active older adults present increased fall risk, as they are more exposed than their sedentary counterparts [19]. It was indicated that modest increases in PA seem to improve overall functional performance, which may represent a positive protective factor for falls. Comparatively, fallers may benefit from fewer minutes of PA than non-fallers. Although the octogenarians with a fall history were insufficiently active, they are prone to achieve considerable functional gains since their adaptive margins are broader [14].

The amount of PA required to impact the HGS and the 5STS must be viewed cautiously. It is unlikely that a limited time (i.e., less than 2 min per day) causes positive impacts on strength and power. Indeed, PA was assessed using the sum of all activities performed in a week, irrespective of its nature or intensity. Thus, the time spent in low-intensity activities (e.g., walking at a self-selected speed) is computed similarly to the time spent in far more vigorous exercises (e.g., a resistance training program). For instance, maintaining a standing posture, walking, and standing from a chair requires low muscle activations (from 30 to 40% of the maximum [20,21,22]). In addition, Rodacki and colleagues identified negligible to low correlations between HGS and lower limb strength, which were considered limited to reflect functional performance in very old adults [23]. On the other hand, regular PA may elicit low muscle activation and cause a positive influence on fall risk, as it may improve walking and balance performance.

Maintaining a physically active lifestyle is relevant as it provides several benefits, such as preserved cognitive and physical functions [24], incremented functional capacity [25], improved balance, muscle strength, and life quality [26]. Physically active oldest-old adults are also more independent and likely to have healthier aging compared to sedentary or insufficiently active counterparts. This is particularly relevant among octogenarians, who are more likely to become frailer and have greater fall risk as they age. Our results are suggestive that the oldest-old adults with more than 300 min week^−1^ are less prone to frailty and falls. These results align with the physical activity guidelines [27] that recommend at least 150–300 min week^−1^ of moderate-intensity aerobic exercises. It has been suggested that sessions as short as 8 min [28] can be beneficial when they include both aerobic and resistance training [29].

## 5. Study Limitations

Our study has several limitations. The first relates to its transversal comparative nature, which requires experimental evidence that PA increments can modulate frailty among oldest-old adults. The second limitation refers to the low number of very active octogenarians present among the participants. The third concerns the PA subjective assessment, which does not consider the nature or intensity of the physical activities performed. Finally, fall assessment relies on memory and may be biased since not all falls may be remembered, even though fall prevalence was within most literature reports.

## 6. Conclusions

It can be concluded that PA is a suitable protective and attenuating factor of the adverse effects of aging octogenarians since individuals with lower levels of PA had higher frailty vulnerability scores and worse performance when compared to their peers with a high level of PA. The present study extends the knowledge about the oldest-old population, especially for health and caring professionals, since small increases in PA may cause physical and functional improvements and reduce frailty and fall risk. Thus, it is suggested that health promotion and fall prevention programs for the oldest-old adults encourage increases in PA to promote healthy aging. Longitudinal studies are required to confirm whether such increases are sufficient to cause positive frailty transitions, improve independence, and reduce the fall prevalence among oldest-old adults.

## Figures and Tables

**Table 1:** Sociodemographic characteristics of all participants (ALL), insufficiently active (IAG), active (AG), and very active (VAG) groups.

Characteristics	ALL (*n* = 239) Mean ± SD	IAG (*n* = 98) Mean ± SD	AG (*n* = 81) Mean ± SD	VAG (*n* = 60) Mean ± SD	*p* Value
	*n* (%)	*n* (%)	*n* (%)	*n* (%)	*n* (%)
**Education level**					0.498
Illiterate	29 (12.1)	12 (12.2)	12 (14.8)	5 (8.3)	
1–8 years	158 (66.1)	66 (67.3)	51 (63.0)	41 (68.3)	
>8 years	24 (10.0)	11 (11.2)	5 (6.2)	8 (13.4)	
Higher education	28 (11.8)	9 (9.3)	13 (16.0)	6 (10.0)	
**Marital status**					0.724
Married	58 (24.3)	26 (26.5)	17 (21.0)	15 (25.0)	
Divorced	8 (3.3)	2 (2.0)	3 (3.7)	3 (5.0)	
Single	17 (7.1)	9 (9.2)	6 (7.4)	2 (3.3)	
Widowed	156 (65.3)	61 (62.2)	55 (67.9)	40 (66.7)	

IAG = Insufficiently Active; AG = Active; VAG = Very Active; BMI = Body Mass Index; PA = Physical Activity level.

**Table 2:** Comparison of physical characteristics, frailty, vulnerability, functional capacity, and falls of all participants (ALL) and between insufficiently active (IAG), active (AG), and very active (VAG) groups.

Characteristics	ALL (*n* = 239) Mean ± SD	IAG (*n* = 98) Mean ± SD	AG (*n* = 81) Mean ± SD	VAG (*n* = 60) Mean ± SD	*p* Value
Age (years)	84 ± 3	84 ± 4	84 ± 3	84 ± 3	0.658
BMI (kg m^−2^)	26 ± 4	26 ± 4	27 ± 4	26 ± 3	0.343
PA (min week^−1^)	209 ± 157	69 ± 44 ^b,c^	221 ± 46 ^a,c^	421 ± 124 ^a,b^	<0.001
5STS (s)	13.97 ± 5.03	15.63 ± 5.16 ^b,c^	13.01 ± 4.55	12.56 ± 4.72	<0.001
TUG (s)	12.23 ± 5.09	13.55 ± 5.72 ^b,c^	11.47 ± 4.00	11.11 ± 4.91	0.002
BBS (points)	48.61 ± 7.68	46.72 ± 8.25 ^c^	49.12 ± 7.11	51.00 ± 6.71	0.001
HGS (kgf)	20.58 ± 6.98	20.26 ± 7.31	20.65 ± 5.87	21.00 ± 7.86	0.843
**Gender**	***n* (%)**	***n* (%)**	***n* (%)**	***n* (%)**	
Female	176 (73.6)	70 (39.8)	59 (33.5)	47 (26.7)	0.620
Male	63 (26.4)	28 (44.4)	22 (34.9)	13 (20.6)	
**Falls**					
Yes	110 (46.0)	67 (68.4) ^b,c^	29 (35.8)	14 (23.3)	<0.001
No	129 (54.0)	31 (31.6) ^b,c^	52 (64.2)	46 (76.7)	
**Frailty Vulnerability (CFVI-20)**					
Low vulnerability	67 (28.0)	17 (17.3) ^b,c^	30 (37.0)	20 (33.3)	0.001
Moderate vulnerability	98 (41.0)	37 (37.8)	31 (38.3)	30 (50.0)	
High vulnerability	74 (31.0)	44 (44.9) ^b,c^	20 (24.7)	10 (16.7)	

IAG = Insufficiently Active; AG = Active; VAG = Very Active; BMI = Body Mass index; PA = Physical Activity; 5STS = Five Times Sit-to-Stand Test; TUG = Time Up and Go; BBS = Berg Balance Score; HGS = Hand Grip Strength; CFVI-20 = Clinical-Functional Vulnerability Index. ^a^ Differ from the IAG; ^b^ Differ from the AG; ^c^ Differ from the VAG.

## Data Availability

Data may be made available upon reasonable request.

## References

[1] Zhang P.D., Lv Y.B., Li Z.H., Yin Z.X., Li F.R., Wang J.N., Zhang X.R., Zhou J.H., Wu X.B., Duan J. (2020). Age, period, and cohort effects on activities of daily living, physical performance, and cognitive functioning impairment among the oldest-old in China. J. Gerontol. Ser. A Biol. Sci. Med. Sci..

[2] Morley J.E., Vellas B., Van Kan G.A., Ankler S., Bauer J.M., Bernabei R., Cesari M., Chumlea W.C., Doehner W., Evans J. (2013). Frailty Consensus: A Call to Action. J. Am. Med. Dir. Assoc..

[3] Moreira N.B., Bento P.C.B., Vieira E.R., da Silva J.L.P., Rodacki A.L.F. (2022). Comparison between the Clinical-Functional Vulnerability and the Frailty Phenotype to Identify Falls in Elderly: A Cross-Sectional Study. Ann. Phys. Rehabil. Med..

[4] Landi F., Abbatecola A.M., Provinciali M., Corsonello A., Bustacchini S., Manigrasso L., Cherubini A., Bernabei R., Lattanzio F. (2010). Moving against frailty: Does physical activity matter?. Biogerontology.

[5] Berkemeyer K., Wijndaele K., White T., Cooper A.J.M., Luben R., Westgate K., Griffin S.J., Khaw K.T., Wareham N.J., Brage S. (2016). The descriptive epidemiology of accelerometer-measured physical activity in older adults. Int. J. Behav. Nutr. Phys. Act..

[6] Moreira N.B., Rodacki A.L.F., Pereira G., Bento P.C.B. (2018). Does functional capacity, fall risk awareness and physical activity level predict falls in older adults in different age groups?. Arch. Gerontol. Geriatr..

[7] Franceschi C., Garagnani P., Morsiani C., Conte M., Santoro A., Grignolio A., Monti D., Capri M., Salvioli S. (2018). The continuum of aging and age-related diseases: Common mechanisms but different rates. Front. Med..

[8] Ramsey K.A., Rojer A.G.M., D’Andrea L., Otten R.H.J., Heymans M.W., Trappenburg M.C., Verlaan S., Whittaker A.C., Meskers C.G.M., Maier A.B. (2021). The association of objectively measured physical activity and sedentary behavior with skeletal muscle strength and muscle power in older adults: A systematic review and meta-analysis. Ageing Res. Rev..

[9] Lustosa L.P., Pereira D.S., Dias R.C., Britto R.R., Parentoni A.N., Souza L., Pereira M. (2011). Tradução e adaptação transcultural do Minnesota Leisure Time Activities Questionnaire em idosos. Geriatr. Gerontol..

[10] Chodzko-Zajko W.J., Proctor D.N., Fiatarone Singh M.A., Minson C.T., Nigg C.R., Salem G.J., Skinner J.S. (2009). Exercise and physical activity for older adults. Med. Sci. Sports Exerc..

[11] de Moraes E.N., do Carmo J.A., de Moraes F.L., Azevedo R.S., Machado C.J., Montilla D.E.R. (2016). Clinical-Functional Vulnerability Index-20 (IVCF-20): Rapid recognition of frail older adults. Rev. Saude Publica.

[12] Moreira B., Bento P.C.B., Vieira E.R., Silva L.P., Rodacki A.L.F. (2022). Association between Domains of the Clinical-Functional Vulnerability Index and Falls History in Older Adults: A Cross-Sectional Study. Int. J. Environ. Res. Public Health.

[13] Sato S., Takeda N., Yamada T., Nakamura M., Nemoto Y., Maruo K., Fukuda Y., Sawada S.S., Kitabatake Y., Arao T. (2022). Physical activity and/or sedentary behaviour and the development of functional disability in community-dwelling older adults in Tsuru, Japan: A prospective cohort study (the Tsuru Longitudinal Study). BMJ Open.

[14] Hortobágyi T., Mizelle C., Beam S., DeVita P. (2003). Old adults perform activities of daily living near their maximal capabilities. J. Gerontol. Ser. A Biol. Sci. Med. Sci..

[15] Zhou J., Liu B., Qin M.Z., Liu J.P. (2021). A prospective cohort study of the risk factors for new falls and fragility fractures in self-caring elderly patients aged 80 years and over. BMC Geriatr..

[16] Barry E., Galvin R., Keogh C., Horgan F., Fahey T. (2014). Is the Timed Up and Go test a useful predictor of risk of falls in community dwelling older adults: A systematic review and meta- analysis. BMC Geriatr..

[17] Bouatois S. (2008). Five Times Sit to Stand Test Is a Predictor of recurrent Falls in Healthy Community Living Subjects aged 65 and Older. J. Am. Geriatr. Soc..

[18] Thorbahn L.D., Newton R.A. (1996). Use of the Berg balance test to predict falls in elderly persons. Phys. Ther..

[19] Dionyssiotis Y. (2012). Analyzing the problem of falls among older people. Int. J. Gen. Med..

[20] Fujiwara K., Toyama H., Asai H., Maeda K., Yaguchi C. (2010). Regular heel-raise training focused on the soleus for the elderly: Evaluation of muscle thickness by ultrasound. J. Physiol. Anthropol..

[21] MacLean M.K., Ferris D.P. (2021). Human muscle activity and lower limb biomechanics of overground walking at varying levels of simulated reduced gravity and gait speeds. PLoS ONE.

[22] Kaneda K. (2019). The features of muscle activity during chair standing and sitting motion in submerged condition. PLoS ONE.

[23] Rodacki A.L.F., Moreira N.B., Pitta A., Wolf R., Filho J.M., Rodacki C.d.L.N., Pereira G. (2020). Is handgrip strength a useful measure to evaluate lower limb strength and functional performance in older women?. Clin. Interv. Aging.

[24] Lin Y.H., Chen Y.C., Tseng Y.C., Tsai S.T., Tseng Y.H. (2020). Physical activity and successful aging among middle-aged and older adults: A systematic review and meta-analysis of cohort studies. Aging.

[25] Ferretti F., Macagnan D., Canei F.C., da Silva M.R., dos Santos M.P.M. (2020). Physical activity level among older adults over 70 years old and very old adults. Fisioter. Mov..

[26] Patti A., Zangla D., Sahin F.N., Cataldi S., Lavanco G., Palma A., Fischietti F. (2021). Physical exercise and prevention of falls. Effects of a Pilates training method compared with a general physical activity program A randomized controlled trial. Medicine.

[27] World Health Organization (2020). WHO Guidelines on Physical Activity and Sedentary Behaviour.

[28] Izquierdo M., Merchant R.A., Morley J.E., Anker S.D., Aprahamian I., Arai H., Aubertin-Leheudre M., Bernabei R., Cadore E.L., Cesari M. (2021). International Exercise Recommendations in Older Adults (ICFSR): Expert Consensus Guidelines. J. Nutr. Health Aging.

[29] Visser D., Wattel E.M., Gerrits K.H.L., van der Wouden J.C., Meiland F.J.M., de Groot A.J., Jansma E.P., Hertogh C.M.P.M., Smit E.B. (2022). Effectiveness and characteristics of physical fitness training on aerobic fitness in vulnerable older adults: An umbrella review of systematic reviews. BMJ Open.

